# Floating norms for individualising the ANB angle and the WITS appraisal in orthodontic cephalometric analysis based on guiding variables

**DOI:** 10.1007/s00056-021-00322-1

**Published:** 2021-07-13

**Authors:** Eva Paddenberg, Peter Proff, Christian Kirschneck

**Affiliations:** Department of Orthodontics, University Hospital Regensburg, 93053 Regensburg, Germany

**Keywords:** Skeletal sagittal class, Lateral cephalograms, Angle class I malocclusion, Orthodontic treatment, Orthodontic diagnostics, Skelettale sagittale Klasse, Fernröntgenseitenbilder, Angle-Klasse-I-Malokklusion, Kieferorthopädische Behandlung, Kieferorthopädische Diagnostik

## Abstract

**Purpose:**

The sagittal skeletal relationship of maxilla and mandible (skeletal class) can generally be determined via lateral cephalograms (ANB angle or Wits appraisal) by comparing measurements to empirical norms based on the respective population mean. However, values differing from these empirical norms also enable a therapeutically desired, normal class I occlusion depending on individual craniofacial pattern, thus requiring floating norms based on guiding variables. As available regression equations consider only few predictor variables and are not up-to-date regarding a contemporary patient collective, the aim of this study was to establish improved and extended regression equations for individualising the ANB angle and Wits appraisal.

**Methods:**

This retrospective, cross-sectional multicentre study was based on 71 Caucasian male and female subjects of any age with normal dental occlusion. We cephalometrically analysed digitised pretreatment lateral radiographs and performed multiple linear regression analyses to identify suitable skeletal predictor variables for individualising the ANB angle and Wits appraisal.

**Results:**

Inter- and intrarater reliability tests showed mostly perfect measurement concordance. Both original regression equations by Panagiotidis/Witt and Järvinen could be updated for a contemporary population with new regression coefficients. The equation for individualising the ANB could be further optimised in its prediction reliability by adding the skeletal predictor variables NL-NSL, NSBa, facial axis (Ricketts) and index (Hasund), whereas the recalculated Wits equation could not be further improved by additional guiding variables.

**Conclusions:**

The improved regression formulae for individualising the ANB angle and Wits appraisal should help to improve the assessment of sagittal skeletal class in clinical orthodontic practice.

## Abstract (de)

**Ziel:**

Die sagittale skelettale Lagebeziehung zwischen Ober- und Unterkiefer (skelettale Klasse) kann im Allgemeinen anhand von Fernröntgenseitenbildern (ANB, Wits-Wert) bestimmt werden, indem Messungen mit empirischen Normen auf der Grundlage des jeweiligen Populationsmittelwerts verglichen werden. Werte, die von diesen empirischen Normen abweichen, ermöglichen im Einzelfall jedoch ebenfalls eine therapeutisch erwünschte, normale Klasse-I-Okklusion in Abhängigkeit vom individuellen kraniofazialen Muster, weshalb fließende Normen erforderlich sind, die auf leitenden Variablen basieren. Da die verfügbaren Regressionsgleichungen nur wenige Prädiktorvariablen berücksichtigen und in Bezug auf ein aktuelles Patientenkollektiv nicht auf dem neuesten Stand sind, bestand das Ziel dieser Studie darin, verbesserte und erweiterte Regressionsgleichungen zur Individualisierung des ANB-Winkels und der Wits-Bewertung zu erstellen.

**Methode:**

Diese retrospektive multizentrische Querschnittsstudie basierte auf 71 kaukasischen männlichen und weiblichen Probanden jeden Alters mit normaler Klasse-I-Okklusion. Wir analysierten digitalisierte Fernröntgenseitenbilder vor kieferorthopädischer Therapie kephalometrisch und führten mehrere lineare Regressionsanalysen durch, um geeignete skelettale Prädiktorvariablen zur Individualisierung des ANB-Winkels und der Wits-Bewertung zu identifizieren.

**Ergebnisse:**

Interrater- und Intrarater-Reliabilität-Tests zeigten größtenteils eine perfekte Messkonkordanz. Beide ursprünglichen Regressionsgleichungen – von Panagiotidis/Witt und von Järvinen – konnten für eine zeitgenössische Population mit neuen Regressionskoeffizienten aktualisiert werden. Die Gleichung zur Individualisierung des ANB konnte in ihrer Vorhersagezuverlässigkeit weiter optimiert werden, indem die skelettalen Prädiktorvariablen NL-NSL, NSBa, Fazialachse (Ricketts) und Index (Hasund) hinzugefügt wurden, während die neu berechnete Wits-Gleichung durch Hinzufügen weiterer leitender Variablen nicht weiter verbessert werden konnte.

**Schlussfolgerungen:**

Die verbesserten Regressionsformeln zur Individualisierung des ANB-Winkels und des Wits-Appraisal sollten dazu beitragen, die Beurteilung der sagittalen skelettalen Klasse in der kieferorthopädischen Praxis zu verbessern.

## Introduction

**Fig. 1:**
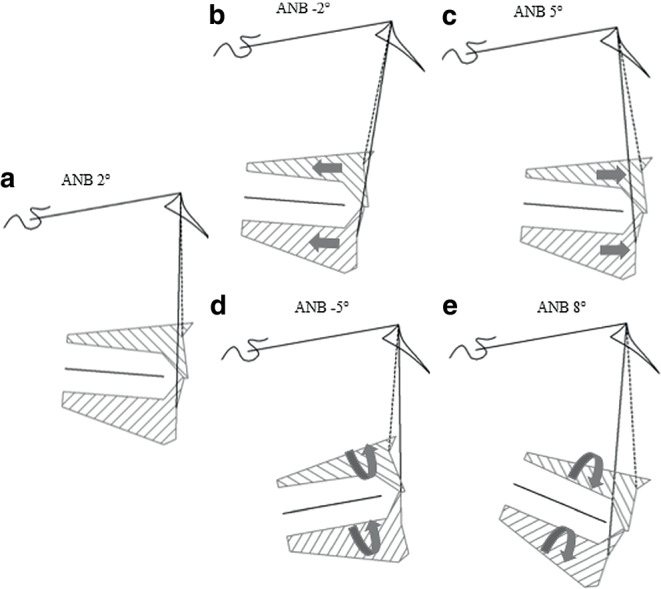
Individual variations of skeletal craniofacial configuration for achieving a normal class I occlusion and corresponding ANB angles corresponding to the ideal “individualised” ANB angles, which can be derived using a regression formula. **a** Upper and lower jaw orthognathic and normally inclined, 2° ANB. **b** Upper and lower jaw retrognathic, −2° ANB. **c** Upper and lower jaw prognathic, 5° ANB. **d** Upper and lower jaw anteriorly inclined, −5° ANB. **e** Upper and lower jaw posteriorly inclined, 8° ANB (Modified after Jacobson (1975) [11]) Individuelle Variationen der skelettalen kraniofazialen Konfiguration, die bei einer Klasse-I-Okklusion vorliegen, sowie die korrespondierenden ANB-Winkel, welche dem idealen, „individualisierten“ ANB-Winkel entsprechen und auch über Regressionsgleichungen zu berechnen sind. **a** Ober- und Unterkiefer orthognath und normoinkliniert, 2° ANB. **b** Ober- und Unterkiefer retrognath, −2° ANB. **c** Ober- und Unterkiefer prognath, 5° ANB. **d** Ober- und Unterkiefer anterior rotiert, −5° ANB. **e** Ober- und Unterkiefer posterior rotiert, 8° ANB. (Mod. nach Jacobson 1975; [11])

**Fig. 2:**
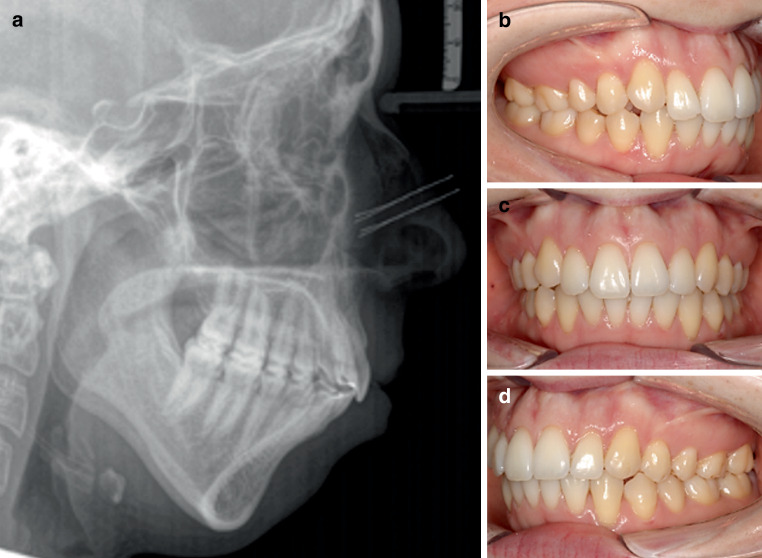
Cephalometric (**a**) and intraoral (**b–d**) situation of a patient with a desired normal class I occlusion after orthodontic treatment despite a supposedly quite pronounced skeletal class II according to ANB and Wits. The lower jaw is retrognathic (SNB 70.4°) and posteriorly inclined (ML-NSL 50.4°), which causes a disharmonious facial type and an increased sagittal distance between points A and B (ANB 10.4°, Wits 8.3 mm). Even though the measured ANB and Wits parameters indicate a distinct skeletal class II, comparing ANB and Wits to individualised floating norms reveals only a small sagittal discrepancy between upper and lower jaw (individualised ANB_Panagiotidis/Witt_ 7.2°, individualised Wits_Järvinen_ 9.8 mm) due to the pronounced deviation of the mandible in vertical direction Kephalometrische (**a**) und intraorale (**b**–**d**) Situation einer Patientin mit der nach abgeschlossener kieferorthopädischer Behandlung erwünschten dentalen Klasse I trotz einer ANB und Wits zufolge vermutlich deutlich ausgeprägten skelettalen Klasse II. Durch die Retrognathie (SNB 70,4°) und posteriore Rotation des Unterkiefers (ML-NSL 50,4°) kommt es zu einem disharmonischen Gesichtstyp und einer vergrößerten sagittalen Distanz zwischen den Punkten A und B (ANB 10,4°, Wits 8,3 mm). Der Vergleich zwischen gemessenem ANB bzw. Wits und deren individuellen Normwerten zeigt eine lediglich geringe sagittale Diskrepanz, also eine skelettale Klasse II, da die Abweichung des Unterkiefers vornehmlich in der Vertikalen durch die posteriore Neigungsdysharmonie auftritt (individualisierter ANB_Panagiotidis/Witt_ 7,2°, individualisierter Wits_Järvinen_ 9,8 mm)

Lateral cephalograms are essential for any orthodontic diagnosis and provide information about skeletal configuration, dental relationship and soft tissue profile against the background of a particular facial type in horizontal and vertical direction [8, 9, 19, 21]. Among others, the skeletal class, defining the sagittal relationship between upper and lower jaw, can be determined by the ANB angle and the Wits appraisal [11], which indicate a dysgnathia, that is a class II or class III relationship, or a neutrobasal class I sagittal relation of maxilla and mandible. To determine the skeletal sagittal class of any patient, usually the measured ANB angle or Wits appraisal are compared to a specific norm value, which can be empirically derived via epidemiological data based on the most commonly encountered ANB or Wits value in the general population. Although these empirical norms are often used as therapeutically desired values, they are not applicable to the majority of subjects, as a normal class I occlusion (or class I skeletal configuration) can also be achieved in a particular patient with ANB and Wits values differing from the population mean. This depends on the individual facial type and craniofacial skeletal configuration (Figs. 1 and 2), as these vary between individuals with a normal class I occlusion. Empirical norms therefore are biased by the population evaluated and not suitable for treatment planning of a particular patient [5]. If the analysis is based on empirical norms irrespective of the individual facial type, this may lead to erroneous treatment and an instable posttreatment situation.

Compared to orthognathic jaw bases and an empirically “normal” ANB of 2° (Fig. 1a), a retrognathic maxilla and mandible regarding the anterior cranial base result in a reduced ANB angle despite a class I skeletal relationship of upper and lower jaw and corresponding class I occlusion (Fig. 1b). Prognathic jaws on the other hand result in an enlarged ANB, despite the jaw relation and occlusion being normal (Fig. 1c). The same is true for an anterior rotation of both maxilla and mandible in relation to SN, which reduces the ANB angle despite the skeletal and dental class I relationship present (Fig. 1d). Consequently, a posterior inclination of both normally related jaws is associated with an increased ANB angle (Fig. 1e). The interpretation of the measured ANB angle in relation to a fixed empirical norm value of 2° is thus not suitable to assess the skeletal class and jaw relation of a particular patient or to derive corresponding treatment decisions. The measured ANB angle and Wits appraisal rather have to be interpreted in relation to a floating norm, which accounts for these interindividual differences in craniofacial phenotype, which can for example be determined by a regression formula as individualised ANB or individualised Wits [16] or by using harmony charts such as the harmony box developed by Hasund and Segner [20].

Very early, Steiner [23] already demonstrated the impact of the ANB angle on acceptable relations between the upper and lower incisors creating floating norms for therapeutically ideal incisor inclination based on the ANB. Segner [20] established floating norms for harmonious combinations of the cephalometric parameters SNA, SNB, ML-NSL, NL-NSL and NSBa. He visualised these graphically with a harmony box corresponding to the individual facial types enabling a class I occlusion. This method allows a distinction between three facial types (ortho-, retro-, prognathic), which were already proposed by Björk [3], as well as between a harmonious and disharmonious facial composition [9, 20]. Furthermore, it enables the identification of the likely causal parameter (sagittal or vertical or both) or jaw (maxilla or mandible or both). Several authors adopted these floating norms for certain populations in terms of age and ethnicity [5, 22, 25]. Another approach to individualise norms is to use multiple regression analyses to derive floating norms based on guiding variables, which was done for individualising the ANB angle [17] and Wits appraisal [13].

The problem with all currently available floating norms and regression equations for individualising the ANB angle and Wits appraisal is the fact that only very few guiding variables were considered resulting in a limited prediction reliability and that these floating norms are not up-to-date regarding the patient collective treated in orthodontics today. Therefore, the aim of this cross-sectional study was to establish new and improved regression formulae for floating norms in terms of an individualised ANB and individualised Wits for determining sagittal skeletal class in clinical orthodontic practice based on guiding variables for a Caucasian population. Furthermore, old regression models for individualising the ANB [17] and Wits appraisal [13] were recalculated and updated based on a contemporary patient collective.

## Materials and methods

**Fig. 3:**
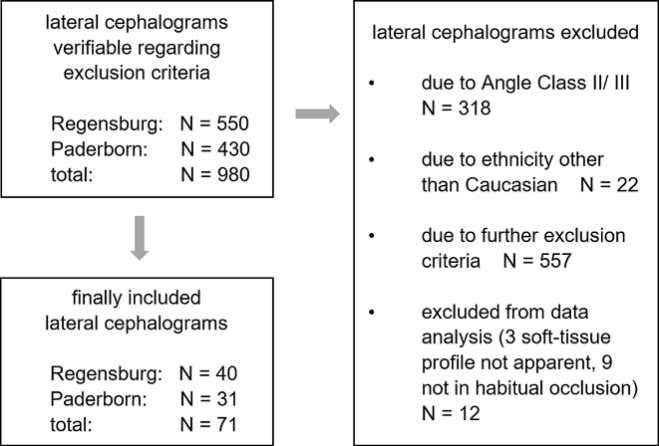
Flow chart of study collective Flow-Chart der Studienteilnehmer

In this retrospective cross-sectional study, the dental and orthodontic records of subjects presenting bilateral Angle class I (normal occlusion after reconstruction) from a German university department (Bavaria, from 2015–2020) as well as an orthodontic specialist practice (North Rhine–Westphalia, from 2019–2020) were screened to establish a patient collective representative of contemporary German subjects. In total, 71 patients aged between 7 and 66 years and equally distributed in terms of gender contributed to the study population. To avoid any bias, subjects with previous or current orthodontic treatment, syndromes or cleft lip or palate and existing or previous craniofacial pathologies (e.g. cancer, condyle hyper-/hypoplasia) or trauma/fractures in the cranial region were excluded. Furthermore, a missing pretreatment digital lateral cephalogram with scale led to exclusion to allow accurate (metric) cephalometric analysis. Subjects with insufficient diagnostic material to determine occlusion were excluded as well as subjects with any ethnicity other than Caucasian. We thus derived regression formulae for determining the individualised ANB angle or Wits appraisal, which respectively enables a normal occlusion in a particular contemporary Caucasian patient (Fig. 3).

**Fig. 4:**
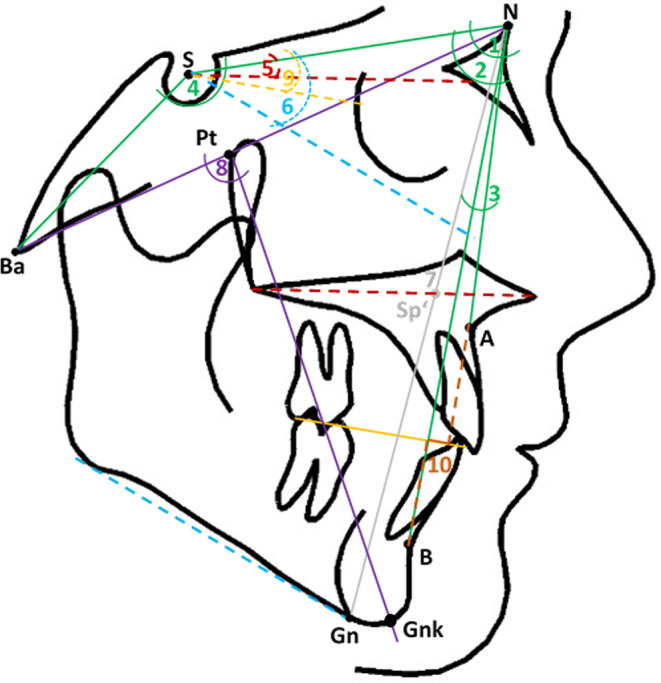
Skeletal cephalometric parameters used as guiding predictor variables for calculating the individualised ANB angle and Wits appraisal in this study: (1) SNA, (2) SNB, (3) ANB, (4) NSBa, (5) NL-NSL, (6) ML-NSL, (7) index (Hasund) = NSp’/Sp’Gn, (8) facial axis (Ricketts), (9) SN-Occl, (10) Wits Skelettale FRS(Fernröntgenseitenbilder)-Parameter, die als leitende Variablen zur Berechnung des individuellen ANB-Winkels und Wits-Appraisal in der Studie verwendet wurden: (1) SNA, (2) SNB, (3) ANB, (4) NSBa, (5) NL-NSL, (6) ML-NSL, (7) Index (Hasund) = NSp’/Sp’Gn, (8) Fazialachse (Ricketts), (9) SN-Occl, (10) Wits

After anonymisation of all patient data directly at the source (anonymised participant number), the pretreatment lateral cephalogram of each patient was imported as lossless TIF file into the software ivoris® analyze pro (Computer konkret AG, Falkenstein, Germany, version 8.2.15.110) and calibrated. Cephalograms were taken at the university department with the devices Orthophos XG 3D ready Ceph and Orthophos SL 2D run with the software Sidexis XG 2.61 and Sidexis 4 version 4.3.1.0 revision 70140, respectively (Dentsply Sirona, Bensheim, Germany). Exposure time was 0.7 s with a voltage of 73 kV and a current of 15 mA. In the orthodontic specialist practice the X‑ray device Vatech PaX‑i Ceph (PCH2500; Vatech Co., Hwaseong-si, Korea) was used with the capture software version 1.0.1.18 and the software byzz 6.2.1 for all images before 17/07/2019 or byzz next version 10.2.89 from 18/07/2019 onwards (orangedental GmbH & Co. KG, Biberach a. d. Riss, Germany). Exposure time was 0.7 s with a current of 10 mA and a voltage of 90 kV for adults and 85 kV for children, respectively. After import, a common digital cephalometric analysis, derived from Segner and Hasund [21], was performed based on 31 reference points and 36 measurements. For this study, only skeletal reference points and lines as well as the derived skeletal parameters were used (Fig. 4; Table 1).

**Table 1:** Skeletal cephalometric parameters used as guiding predictor variables for calculating the individualised ANB angle and Wits appraisal in this study Skelettale FRS(Fernröntgenseitenbilder)-Parameter, die als leitende Variablen zur Berechnung des individuellen ANB-Winkels und Wits-Appraisal verwendet werden

Parameter	Skeletal cephalometric variable	Definition (empirical norm)
1	SNA	Angle between sella, nasion and point A (81.0 ± 2.0°)
2	SNB	Angle between sella, nasion and point B (79.0 ± 2.0°)
3	ANB	Angle between point A, nasion and point B (2.0 ± 2.0°)
4	NSBa	Angle between nasion, sella and basion (130.0 ± 6.0°)
5	NL-NSL	Angle between lines NL and NSL (8.5 ± 3.0°)
6	ML-NSL	Angle between lines ML and NSL (32.0 ± 2.0°)
7	Index (Hasund)	(NSp’/Sp’Gn) × 100 (80.0 ± 9.0%)
8	Facial axis (Ricketts)	Angle between lines NBa and PtGnk (90.0 ± 3.0°)
9	SN-Occl	Angle between lines NSL and Occl (14.5 ± 2.0°)
10	Wits	Distance between a perpendicular from Occl through point A and a perpendicular from Occl through point B (♀ 0.0 ± 1.0 mm, ♂ −1.0 ± 1.0 mm; > 0, if A ventral B)

Based on available pretreatment plaster casts corresponding to the lateral cephalograms, we determined the occlusion according to Angle [2], differentiating between classes I, II/1, II/2 and III in premolar widths. This allowed the identification of subjects with a bilateral class I occlusion and exclusion of subjects with a class II or III occlusion for study purposes. Ethnicity was determined based on visual examination of available pretreatment extraoral photographs and only Caucasian patients were included.

Statistical analysis was conducted using the software IBM® SPSS® Statistics 20 (IBM, Armonk, NY, USA). Prior to the main study, interrater and intrarater reliability of cephalometric analyses as well as determination of Angle class and ethnicity were tested using the Lin concordance correlation coefficient (CCC) for scalar-metric and Cohen’s kappa (κ) for categorical variables [1, 14, 15]. For this purpose, the respective records of 50 randomly selected subjects were evaluated twice and by two independent examiners with a time interval of at least 2 weeks between assessments.

For individualisation of the ANB angle and the Wits appraisal multiple linear regression analyses were performed with ANB and Wits as target variables including all eligible skeletal cephalometric parameters as potential predictor (guiding) variables. These showed a perfect intrarater reliability (CCC > 0.9) without collinearity (Variance Inflation Factor [VIF] < 10, tolerance > 0.1) and contributed significantly to predicting the individualised ANB angle or Wits (one sample t‑test against zero), thus achieving the largest possible and significant (ANOVA) coefficient of determination R^2^ corresponding to prediction reliability, which was assessed according to Cohen’s classification [4].

Furthermore, the original equations of Panagiotidis and Witt [17] and Järvinen [13] were recalculated based on the study collective. The multiple linear regression was performed using only the predictor variables the original authors used, to assess their validity and to possibly update these formulae to better represent the contemporary orthodontic patient collective assessed in this study. The significance level (α-error) was set at *p* ≤ 0.05.

## Results

Initially, patient records of 980 subjects were screened considering male and female subjects of all ages, ethnicities and malocclusions. Finally, the study population included 71 Caucasian subjects with Angle class I (Fig. 3). The average age was 19.3 years with ages ranging from 7.2 to 66.6 years. Descriptive statistics of sociodemographic data showed an even distribution of participants both in terms of gender (33 women, 38 men) and origin (40 from the university department, 31 from the orthodontic specialist office). Inter- and intrarater reliability of the determination of ethnicity and Angle class proved to be very good (κ ≥ 0.888). Likewise, all cephalometric parameters showed a perfect intrarater concordance for the measurements (CCC ≥ 0.9), except for NL-Occl (°), ML-Occl (°) and Wits (mm), and an almost perfect interrater concordance (CCC ≥ 0.8 and 95% confidence interval [CI] CCC ≥ 0.8), except for Wits (mm), NL-NSL (°), SN-Occl (°), NL-Occl (°) and ML-Occl (°).

First, recalculating the regression equation of Panagiotidis and Witt [17] for the individualised ANB based on the study collective yielded new coefficients and an improved goodness-of-fit of the regression model compared to the available equation, thus optimising prediction reliability (R^2^). A remaining 42.2% of variance is left unexplained by the guiding predictor variables SNA and ML-NSL, which significantly predict the individual ANB: *F*(2, 68) = 48,847; *p* < 0.001.

$$\mathrm{ANB}(\text{indiv}.)=-45.359+0.493\times \mathrm{SNA}+0.251\times \text{ML-NSL }(\text{corrected }\mathrm{R}^{2}=0.578)$$

Next, we searched for further skeletal cephalometric parameters as guiding variables for individualising the ANB angle. Apart from the parameters SNA and ML-NSL initially included by Panagiotidis and Witt [17], the four additional variables NSBa, NL-NSL, index (Hasund) and facial axis (Ricketts) were identified to significantly contribute to the prediction of the individualised ANB: *F*(6, 64) = 26.917, *p* < 0.001. The corresponding regression model has a corrected R^2^ of 0.690, thus maximising prediction reliability of the individualised ANB compared to using the classical formula of Panagiotidis and Witt based only on the two predictor variables SNA and ML-NSL.

$$\mathrm{ANB}(\text{indiv}.)=-41.669+0.567\times \mathrm{SNA}+0.11\times \text{ML-NSL }+0.114\times \text{NSBa }+0.132\times \text{NL-NSL }+0.062\times \text{index }\hbox{- }0.289\times \text{facial axis }(\text{corrected }\mathrm{R}^{2}=0.690).$$

Subsequently, also the original formula for individualising the Wits appraisal by Järvinen [13] was recalculated based on the study collective. This resulted in an optimisation of the goodness-of-fit of the regression formula compared to the original equation for the contemporary orthodontic patient collective.

$$\text{Wits }(\text{indiv}.)=57.510+1.526\times \mathrm{ANB}\hbox{ - }0.634\times \mathrm{SNA}\hbox{ - }0.666\times \text{SN-Occl }(\text{corrected }\mathrm{R}^{2}=0.976)$$

The corrected R^2^ of 0.976 indicates that almost all variance can be explained by the guiding predictor variables ANB, SNA and SN-Occl, as originally proposed by Järvinen. These contribute significantly to the prediction of individual Wits: *F*(3, 67) = 952.650, *p* < 0.001.

Despite the goodness-of-fit of the recalculated original equation of Järvinen being excellent, we attempted to also supplement Järvinen’s regression equation for the Wits appraisal with additional skeletal guiding variables to further improve the model’s goodness-of-fit. We could identify two further predictor variables, which contributed significantly, but only very little to the regression model (ML-NSL and index), as evidenced by the negligible regression coefficients, thus achieving a corrected R^2^ of 0.984: *F*(5, 65) = 849.818, *p* < 0.001.

$$\text{Wits }(\text{indiv}.)=57.853+1.572\times \mathrm{ANB}\hbox{ - }0.664\times \mathrm{SNA}\hbox{ - }0.639\times \text{SN-Occl }\hbox{ - }0.03\times \text{ML-NSL }+0.03\times \text{index }(\text{corrected }\mathrm{R}^{2}=0.984)$$

However, this almost perfect goodness-of-fit represents only an increase of 0.8% compared to the original formula based on only ANB, SNA and SN-Occl as predictor variables.

## Discussion

The aim of this retrospective study was to establish floating individual norms for determining the sagittal skeletal class of an individual patient using the ANB angle or Wits appraisal based on guiding variables to optimise predictive reliability in orthodontic treatment planning based on cephalometric diagnosis. For this purpose, we recalculated existing regression models of Panagiotidis and Witt [17] and Järvinen [13] for individualising the ANB angle and Wits appraisal respectively and searched for further skeletal cephalometric parameters as guiding predictor variables to further improve the prediction reliability creating new regression equations.

A data pool representative of the contemporary Caucasian population in Central Europe was generated by collecting patient records in two locally distinct areas of Germany, improving generalisability of the results. Due to the homogeneous distribution of the study population with regard to origin and gender and due to the wide age range considered, the regression equations should be applicable to typical orthodontic subjects within Germany. Since only the Caucasian ethnicity was considered, applicability for other ethnic groups is likely, but could not be assessed in this study.

The mostly perfect concordance of interrater and intrarater reliability for both categorical and scalar-metric variables indicated reproducible measurements. Among scalar-metric variables, substantial concordance of interrater and intrarater reliability could only be demonstrated for the Wits appraisal and NL-NSL. Regarding NL-NSL, reduced reproducibility could be explained by the difficulty of identifying Spa as the anterior reference point of NL, which can cause different definitions of the spinous plane across several raters. The Wits appraisal uses the occlusal plane as a reference line, which also is often defined differently across raters due to the difficulty of identifying dental reference points.

In this study, the data pool reflects a contemporary Central European population compared to previous investigations of Panagiotidis and Witt [17] and Järvinen [13]. In the past, several authors found (minor) differences in cephalometric values or craniofacial patterns in terms of population [5, 6, 18, 22, 25]. In addition, since the original regression formula for the individualised Wits appraisal of Järvinen [13] considered subjects with all Angle classes, we could increase its precision and validity by taking into account only subjects with class I occlusion. This way, the derived individual norms are sure to enable a desired class I occlusion therapeutically. Furthermore, the new formulae incorporate additional guiding variables, which resulted in a better prognosis of individual norms. Whereas the prediction reliability of the ANB angle could be increased by 11.2%, that of the original Wits formula was only improved by 0.8%.

As Moyers and Bookstein [16] explained, the ANB angle has not only two degrees of freedom to express sagittal relationship between points A and B, but six. Hence the angle is influenced by the relative position of each point, which in turn correlate with the craniofacial patterns of the individual. Panagiotidis and Witt [17] and Järvinen [12] showed the impact of the parameters SNA and ML-NSL on ANB, which was also the case in the publications by Segner [20] and Hasund [9]. Since these parameters affect each other during growth, they contribute to facial type and hence to ANB. Among the newly added skeletal predictor variables the direction of growth was considered by incorporating facial axis. In the past, it has been shown that many parameters are affected by growth, such as vertical variables, mandibular rotation, SNA and SNB [7], which all influence the ANB [10]. Thus, including the facial axis according to Ricketts as predictor variable expressing the growth pattern, seems logical. By embedding the index according to Hasund, another vertical parameter was taken into account, which demonstrates the ANB’s dependence on morphological structures other than SNA and SNB. With the NL-NSL as a guiding variable for maxillary inclination, another vertical parameter was considered, which correlates with other parameters determining the facial type, such as SNA, SNB, ML-NSL and NSBa, and therefore also with the ANB angle [9, 20]. Lastly, NSBa was included, determining facial type being associated with the degree of prognathism and inclination of the maxilla and mandible [21]. Except for SNB, all parameters listed in the harmony box of Segner [20] (SNA, ML-NSL, NL-NSL, NSBa) were identified as guiding predictor variables in the new extended regression equation for the individualised ANB.

Comparing the original and the extended new regression formula for the individualised ANB, not only additional guiding variables were added, but also different regression coefficients were obtained. Whereas SNA exerts a stronger (and the strongest) influence in the new formula, mandibular rotation and the constant became less important. In addition, all new parameters but the index affect ANB more than ML-NSL.

Prediction reliability of the original regression equation for the individualised Wits appraisal according to Järvinen was improved by recalculation. With a corrected R^2^ of 0.976, the original formula based on ANB, SNA and SN-Occl was already quite reliable in predicting the individual Wits. As ANB is a strong indicator of skeletal class, just as the Wits appraisal, its distinct impact in the formula seems logical. In addition, SNA is an important guiding variable, because it correlates with facial type, which affects skeletal class. With SN-Occl, the original and extended formulae consider the occlusal plane, which due to geometry affects Wits. Although in the newly established formula two further predictor variables, ML-NSL and index, were identified as significant guiding variables, there was no clinically relevant improvement of goodness-of-fit with both variables contributing only very little to the individualised Wits. Thus, the usage of the newly calculated original formula based on only ANB, SNA and SN-Occl is recommended for clinical practice. The Wits appraisal seems to be less dependent on other craniofacial skeletal structures than the ANB angle. However, using the occlusal plane as reference could be problematic due to its reduced interrater reliability in measurement and it being affected by the dentition.

Among the new predictor variables are also some considering the growth pattern. A limitation for growing subjects is the lack of knowledge about the extent of individual growth to be expected [24]. Furthermore, functional aspects could be a potential confounder. With the cross-sectional study type, we could illustrate an association between ANB/Wits, i.e. skeletal class and certain skeletal parameters expressing individual craniofacial patterns. However, we could not identify a cause–effect relation between individual norms and guiding variables [10].

## Conclusions

We established new and improved regression formulae for floating norms in terms of an individualised ANB angle and individualised Wits appraisal for determining the sagittal skeletal class in clinical orthodontic practice based on guiding variables. The original regression equation for the individualised Wits appraisal by Järvinen [13] could be optimised by recalculation, but an extension of the existing model by adding further skeletal predictor variables did not yield a significant improvement of goodness-of-fit, which was very high in the first place. In contrast, the original regression equation for the individualised ANB by Panagiotidis and Witt [17] could not only be updated for a contemporary orthodontic patient collective by calculating new regression coefficients, but improved in its prediction reliability by adding the additional four skeletal guiding predictor variables NL-NSL, NSBa, facial axis (Ricketts) and index (Hasund).
